# Use of Intracorporeal Durable LVAD Support in Children Using HVAD or HeartMate 3—A EUROMACS Analysis

**DOI:** 10.3390/jcdd10080351

**Published:** 2023-08-17

**Authors:** Martin Schweiger, Hina Hussein, Theo M. M. H. de By, Daniel Zimpfer, Joanna Sliwka, Ben Davies, Oliver Miera, Bart Meyns

**Affiliations:** 1 Department of Congenital Cardiovascular Surgery, Pediatric Heart Center, University Children’s Hospital Zurich, 8032 Zurich, Switzerland; 2 Children’s Research Center, University Children’s Hospital Zurich, 8032 Zurich, Switzerland; 3 Quality and Outcomes Research Unit, University Hospital Birmingham, Birmingham B15 2TH, UK; hwaheed1@hotmail.co.uk; 4 EUROMACS, EACTS House, Windsor SL4 lEU, UK; 5 Department for Heart Surgery, Medical University Graz, Graz A-8010, Austria; 6 Department of Cardiac Surgery, Transplantology and Vascular Surgery, Silesian Center for Heart Diseases, 41-800 Zabrze, Poland; 7 Royal Children’s Hospital, Melbourne 3052, Australia; bdavies.1@protonmail.com; 8 Department of Congenital Heart Diseases—Pediatric Cardiology, Deutsches Herzzentrum der Charité, 13353 Berlin, Germany; oliver.miera@dhzc-charite.de; 9 Department of Cardiac Surgery, University Hospitals Leuven, Herestraat 49, 3000 Leuven, Belgium; bart.meyns@uzleuven.be

**Keywords:** pediatric heart failure, durable intracorporeal LVAD in children, HeartMate 3, HVAD

## Abstract

**Purpose:** The withdrawal of HVAD in 2021 created a concern for the pediatric population. The alternative implantable centrifugal blood pump HeartMate 3 has since been used more frequently in children. This paper analyses the outcome of children on LVAD support provided with an HVAD or HM3. **Methods:** A retrospective analysis of the EUROMACS database on children supported with VAD < 19 years of age from 1 January 2009 to 1 December 2021 was conducted. All patients with an LVAD and either an HVAD or HM3 were included. Patients with missing data on VAD status and/or missing baseline and/or follow up information were excluded. Kaplan–Meier survival analysis was performed to evaluate survival differences. Analyses were performed using Fisher’s exact test. **Results:** The study included 150 implantations in 142 patients with 128 implants using an HVAD compared to 28 implants using an HM3. Nine patients (6%) needed temporary right ventricular mechanical support, which was significantly higher in the HM3 group, with 25% (*p*: 0.01). Patients in the HVAD group were significantly younger (12.7 vs. 14.5 years, *p*: 0.01), weighed less (45.7 vs. 60 kg, *p*: <0.000) and had lower BSA values (1.3 vs. 1.6 m^2^, *p*: <0.000). Median support time was 204 days. Overall, 98 patients (69%) were discharged and sent home, while 87% were discharged in group HM3 (*p*: ns). A total of 123 children (86%) survived to transplantation, recovery or are ongoing, without differences between groups. In the HVAD group, 10 patients (8%) died while on support, whereas in 12% of HM3 patients died (*p*: 0.7). **Conclusions:** Survival in children implanted with an HM3 was excellent. Almost 90% were discharged and sent home on the device.

## 1. Background

Survival of children undergoing left ventricular assist device (LVAD) implantation remains challenging due to the fact that there are few suitable devices for children [1,2,3] and the outcome is age-dependent [1,4,5,6,7]. Despite survival, quality of life while on support plays an important role [8]. This includes home discharge. Over ten years ago, successful home discharge of children with intracorporeal (IC) LVADs was reported [9,10]. Since then, the use of IC-LVADs, especially in children weighing more than 15 to 20 kg, has increased [11], and there seems to be a consensus among pediatric VAD centers with respect to the use of IC-LVADs in these patients [12]. According to well-known North American and European VAD databases, today, around 40% of pediatric patients on VAD support are discharged and sent home or to rehabilitation [13], whereas in those with IC-LVADs the discharge rate is almost half [14]. One of the most used devices is the HeartWare ventricular assist device (HVAD) (Medtronic, Minneapolis, MN, USA). On 3 June 2021, Medtronic announced the withdrawal of the HVAD from the global market [15], a decision that had immediate ramifications in the world of pediatric durable VAD therapy, as the HVAD had been commonly used for IC-LVAD therapy until that time [16].

Fortunately, the HeartMate 3 (HM3) (Abbott, Chicago, IL, USA) was a readily available alternative, having received pediatric approval from the FDA in 2020, despite having historically lagged behind HVAD use in the pediatric sphere.

Aim of the study: This analysis investigates differences in age, size and weight in patients < 19 years of age using an HVAD or an HM3.

## 2. Methods

This is a retrospective analysis of the EUROMACS database. A submitted study proposal was reviewed and approved by the EUROMACS Committee. All patients under 19 years of age provided with an LVAD were included. Patients with missing data on the status of the implanted device were excluded, as well as patients for whom missing baseline and/or followup information was still missing if the individual center was contacted and asked for the missing data but did not respond. Patients with implanted durable biventricular VADs were excluded to keep the cohort as uniform as possible.

Data were analyzed for patients enrolled between the launch of the database (1 January 2009) until 1 December 2021. The primary independent variable of interest was survival after LVAD implantation. The patients were classified into the GVAD or HM3 group according to the presence of an implanted device.

### 2.1. Study Variables

The primary variables were age, weight and body surface area (BSA) at the time of implantation, as well as survival—either to transplantation or recovery. Other variables included reason for death and the time of support and transplantation. Kaplan–Meier survival analysis was performed to evaluate survival differences between children on different durable IC-LVADs.

### 2.2. Statistical Analysis

Data are presented as mean ± standard deviation (SD) or frequency with percentage. Comparisons were performed using Student’s *t*-test. Statistical analysis was performed using IBM SPSS Statistics, Version 24.0 (Armonk, NY, USA: IBM Corp.). Comparisons were performed using Student’s *t*-test or the Mann–Whitney *U* test. Qualitative variables were analyzed by Fisher’s exact test. A 2-sided *p* value of 0.05 or lower was considered to indicate statistical significance. Tables were constructed using MS Excel.

## 3. Results

### 3.1. Study Population

In the observed time period, 177 data entries were found in the database. A total of 27 patients were excluded due to missing data. Important baseline variables like age, weight and BSA were missing for two patients, and no (basic) follow-up data were available for eight patients, such as whether the patient was still alive, still on VAD support or already transplanted (even if center was contacted). Another 17 patients had to be excluded because they received durable biventricular support after temporary right ventricular mechanical support.

In this study, 150 implantations in 142 patients (55 female and 87 male) were included. The median age was 13.1 years, ranging from 3 to 18 years. Primary diagnoses were any form of cardiomyopathy (CMP). Most of the patients had a form of dilated CMP, and only seven patients had either restrictive (two) or hypertrophic (seven) CMP. Implantation was performed in the 5% of patients suffering from congenital heart disease (CHD) (Table 1). At the time of implantation, almost half of the patients were categorized as INTAMACS profile 2 (49%). Still, over 15% were implanted with a durable LVAD in a ‘crash and burn’ situation (INTERMACS profile 1).

Twenty-seven children were on extracorporeal life support, and 78% of all patients were supported on inotropes prior to VAD placement. Over 80% of all children were thought to be bridged to transplantation, and a minority of less than 3% were on permanent support. Baseline characteristics are summarized in Table 2.

**Figure 1:**
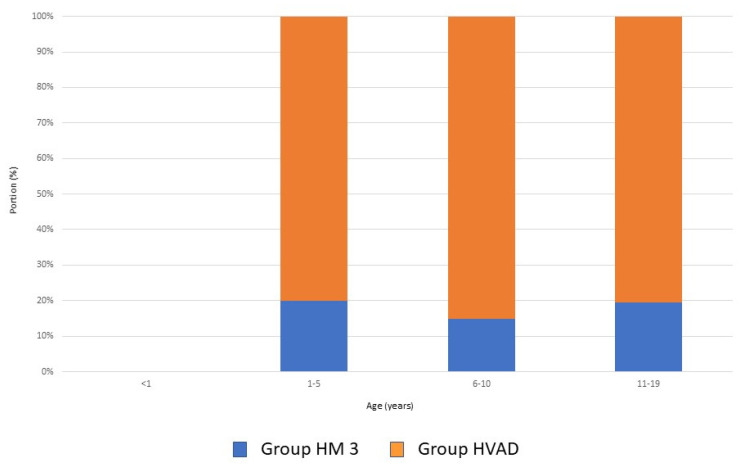
Distribution of devices according to age group.

**Figure 2:**
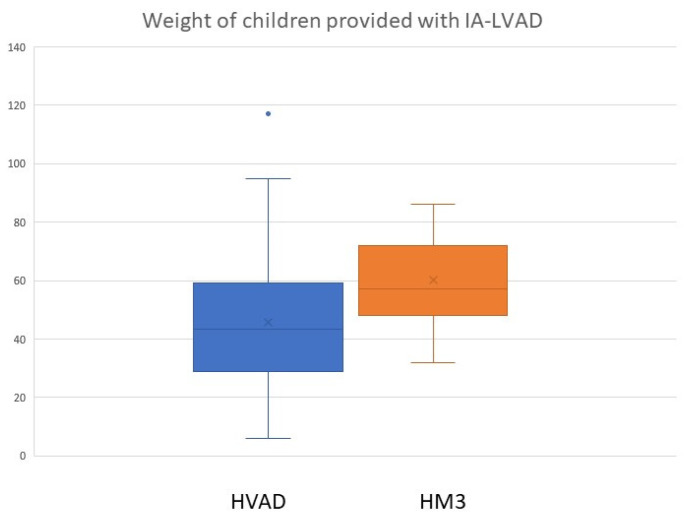
Differences in body weight between the HVAD and HM3 groups.

The distribution of devices according to age group is depicted in Figure 1. Out of 150 implantations, 122 were performed using an HVAD in 118 patients (49 ♀, 69 ♂), forming the HVAD group. The other 28 implants were performed in 24 patients (6 ♀, 18 ♂) using an HM3, forming the second group (HM3 group). Patients in the HVAD group were significantly younger, with a median age of 12.8 (range: 3–18; *p*: 0.01) and had a median weight of 45.7 kg (range: 6–117; *p*: <0.005) and a BSA of 1.3 m^2^ (range: 0.53–2.35; *p*: <0.001). Children provided with a HM3 had a median age of 14.5 years (range: 3–18), a median weight of 60 kg (range: 32–86) and a BSA of 1.6 m^2^ (range: 1.1–2.1). Differences in body weight are shown in Figure 2.

Overall, five children under the age of 6 years received an intracorporeal LVAD, all of which were HVADs. In this analysis, the youngest child provided with an HM3 was 9 years old and weighted 32 kg. In this patient, the LVAD was explanted due to myocardial recovery 333 days after implantation. Only one child weighed less than 5 kg, who received an intracorporeal LVAD (HVAD), while all patients with a weight between 10 and 20 kg received an HVAD (n:13). Two patients weighing between 20 and 40 kg received an HM3. All children suffering from CHD received an HVAD.

### 3.2. Outcomes

**Figure 3:**
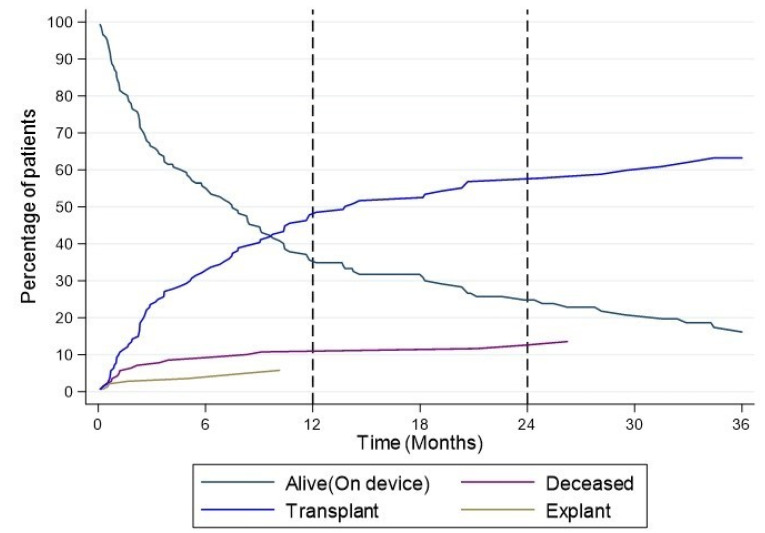
Competing outcome curves for the whole study population.

A total of 123 children (86%) survived to transplantation or recovery or have ongoing cases (Figure 3). Median support time was 204 days, ranging from 3 days to 8.4 years, and did not differ between groups. Median ICU stay was 25 days for HVAD patients and not significantly longer than the 20 days for HM3 patients. Nine patients (6%) needed temporary right ventricular mechanical support, which was significantly higher in the HM3 group, at 25% (*p*: 0.01). Ninety-eight patients (69%) were discharged and sent home, while 87% were discharged in the HM3 group, which did not reach significance.

**Figure 4:**
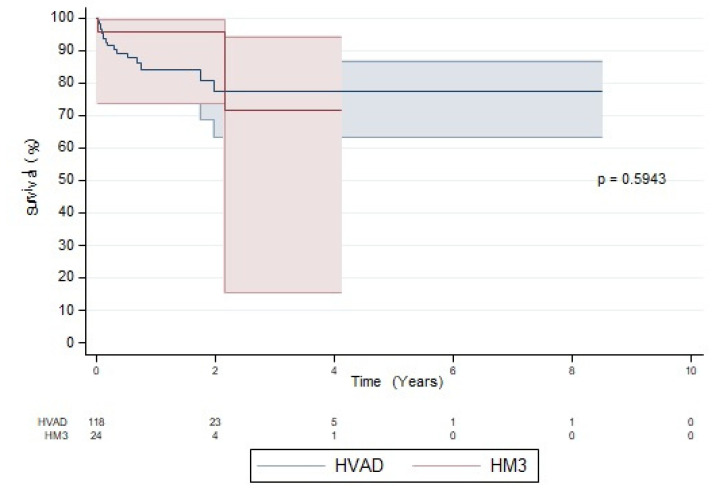
Kaplan–Meier analysis of survival of patients with HeartMate 3 and HeartWare ventricular assist devices.

There were no differences between groups regarding survival (Figure 4), transplantation or recovery. In the HVAD group, 10 patients (8%) died while on support, whereas in the HM3 group, 3 patients (12%) died (*p*: 0.7). Multiorgan failure (including infection/sepsis) and CVA were the most common reasons for death (Table 3). In the study, 91 (61%) patients were transplanted (76 vs. 15; *p*: 0.4) after a median waiting time of 295.6 days on VAD support (296 vs. 298; *p*: 0.9). Overall, 14 pump replacements were performed: 10 in the HVAD group and 4 in the HM3 group (*p*: 0.1).

## 4. Discussion

The need for and utilization of both short-term and long-term pediatric devices are rapidly growing [2,14,17,18]. Compared to ECMO, VAD use in children awaiting heart transplantation has been shown to be associated with improved post-transplant survival [19]. In light of the increasing waiting times to receive a suitable donor organ, especially in Europe, the advantages of IC-LVADs have become obvious. Nowadays, it is recommended to use intracorporeal VADs in children over 15 to 20 kg if suitable [12,20].

In the pediatric MCS community, the announcement of the withdrawal of one pf the most frequently used FDA-approved durable VADs had immediate ramifications [16]. While at that time, the HM3 was enjoying majority market share in the adult world of durable mechanical circulatory support (MCS), experience of this system in children was limited; the pediatric MCS community was obliged to rapidly consider the implications of important differences in device size, profile and weight that may influence use in pediatric patients.

The aim of this study was to understand if there are important differences in terms of age, size, weight and indication between the HVAD and HM3 devices that have influenced their use in pediatric patients. This might help to understand the extent to which surgeons and clinicians will have to adapt their strategy using the HM3 device instead of the HVAD device.

EUROMACS represents one of the largest databases on children with durable VAD support, publishing reports on a biannual basis [4,13,21]. This study found no differences in terms of indications and time point for VAD placement between the groups. The main cardiac diagnosis for VAD placement remains any form of CMP.

While there was no reported implantation of an HM3 device in a child suffering from CHD in this study, others have reported successful implants in children and adults suffering from CHD [22]. In the third Paedi-EUROMACS report, the youngest patient provided with an HM3 was 14 years old, with a weight of 39 kg [4]. According to our analysis, we can report that the youngest patient was 9 years old, with a weight of 32 kg and a body surface area of 1.1 m^2^. Data from the ACTION networks show that the HM3 has been implanted in children as young as 8 years old (BSA, 0.78 m^2^; weight, 19 kg) [22].

Although in this study, we found significant differences in age, weight and BSA between the two groups, we see a trend of surgeons adapting to the size difference between the devices via surgical modification for safe use in children under 10 years of age [23,24] or for implantation in restrictive cardiomyopathy [25]. As already pointed out, the HM3 is slightly bulkier and heavier (200 g vs. 160 g), so many surgeons choose to open the pericardium and pleural space to give the body of the HM3 device enough space. Some surgeons reinforce the pocket with a Gore-Tex membrane, while others perform a pexy from the device to the ribs. Most surgeons wrap the uncovered outflow graft with a Gore-Tex membrane or graft to protect the re-entry during transplantation.

These approaches seem to be supported by the survival rate reported in this study, with excellent survival rates to transplantation when using the HM3.

There were no differences regarding pump exchanges. This finding has to be interpreted with caution due to the fact that no data on anticoagulation were analyzed and because there are known differences between anticoagulation protocols in Europe, even when using the same device [26]. In a recently published study, apixaban, together with low-dose aspirin, was safe and effective in children supported with an HM3 device [27].

One of the surprising findings of this analysis was a higher rate of temporary mechanical right ventricular support (RVAD) in patients with an HM3 device. Patient characteristics, especially those on pre-VAD ECMO support or inotropic support, with cardiac arrest, on dialysis or with an INTERMACS profile, were comparable. This result may be a statistical aberration due to the (relatively) low number of patients in the HM3 group. The analysis from the ACTION network reports a lower rate of temporary RVAD support in children with HM3 implants. Differential flow and pressure-volume loop characteristics between the two pumps must also be taken into consideration. Each LVAD has its own unique HQ curve, meaning that the HQ relationship varies between the HM3 and HVAD groups, which may affect right ventricular performance. Detailed HQ curves for the HM3 were previously published in [28]. Further studies are needed to either confirm or refute the findings observed in this study on RVF.

The percentage of home discharge was especially high in the children provided with an HM3 (~90%). This result might be attributed to the fact that most patients provided with an HM3 were teenagers. In these patients, home discharge is easier compared to smaller children, but it underlines the trend that home discharge of children on durable IC-LVAD support has become routine.

Only a few patients suffering from CHD were included in the study. Nevertheless, it has to be mentioned that both devices have been used successfully in patients with a single ventricle [29] especially in failing Fontan patients [30,31,32,33] with cardiac transplantation years after HM3 implantation [34]. Implantation in other forms of CHD is mentioned above, as previously published in [35,36].

### Limitations

Besides its retrospective character and limitations of database analysis, the number of HM3 implants was relatively small compared to the control group in this study. This is also explained by the fact that the first HVAD implant included in this analysis occurred in October 2009, whereas the first report of HM3 placement was from 2015.

## 5. Conclusions

While patients implanted with an HVAD were younger and had lower BSA values and body weights, no differences in outcome were observed. Given the lack of alternatives for durable intracorporeal LVADs, there seems to be a trend of the pediatric MCS community adapting to the HM3 by using it in smaller children. While this study provides a first analysis, given the buildup of pediatric data submissions in the EUROMACS database, it is advised to perform a follow-up when HM3 implant levels and experience have increased, specifically in smaller children.

## Figures and Tables

**Table 1:** Cardiac diagnoses of patients. CMP: cardiomyopathy; DCMP: dilated cardiomyopathy; RCMP: restrictive cardiomyopathy; HOCMP: hypertrophic cardiomyopathy; CHD: congenital heart disease.

		Entire Cohort	HVAD Group	HM3 Group
Patients		142	118	24
Any form of CMP, n (%)		132 (93%)	109 (92%)	23 (96%)
	DCMP	123 (87%)	102 (86%)	21 (88%)
	RCMP	2 (1%)	2 (2%)	0
	HOCMP	7 (5%)	5 (4%)	2 (8%)
CHD, n (%)		7 (5%)	7 (6%)	0
Unknown, n (%)		3 (2%)	2 (2%)	1 (4%)

**Table 2:** Baseline characteristics of patients.

	Overall	HVAD	HM3	*p* Value
**Age (years)**				0.01
Median (range)	13.1 (3–18)	13 (3–18)	15 (9–18)	
Mean ± SD	13.0 ± 3.3	12.8 ± 3.1	14.5 ± 2.7	
**Age categories (years), n (%)**				
1–5 y	5 (3.3)	4 (3.3)	-	
6–10 y	27 (18.0)	23 (18.9)	1 (14.3)	
11–19 y	118 (78.7)	95 (77.9)	24 (82.1)	
**Weight, n (%)**				0.005
<5 kg	1 (0.7)	1 (0.9)	-	
5–9 kg	-	-	-	
10–20 kg	13 (9.2)	13 (11.0)	-	
21–40 kg	41 (28.9)	39 (33.0)	2 (8.3)	
41–70 kg	64 (45.1)	50 (42.4)	14 (58.3)	
>71 kg	23 (16.2)	15 (12.6)	8 (33.3)	
**Body surface area (m^2^)**				0.001
Median (range)	1.4 (0.53–2.5)	1.3 (0.53–2.5)	1.6 (1.1–2.1)	
**Sex, n (%)**				0.130
Male	87 (61.3)	69 (58.5)	18 (75.0)	
Female	55 (38.7)	49 (41.5)	6 (25.0)	
**Total bilirubin levels (mg/dL)**				0.582
Median (range)	0.7 (0–25)	0.7 (0–25)	1.1 (0–9)	
Mean ± SD	1.4 ± 2.7	1.3 ± 2.9	1.7 ± 2.0	
**Creatinine (mg/dL)**				0.635
Median (range)	68 (16–8079)	67 (20–8079)	69 (16–202)	
Mean ± SD	145.4 ± 740.9	161.6 ± 826.5	79.2 ± 36.7	
**Primary diagnosis, n (%)**				0.611
DCMP	121 (85.2)	100 (84.8)	21 (87.5)	
RCMP	2 (1.4)	2 (1.7)	-	
CHD	7 (4.9)	7 (5.9)	-	
CMP	2 (1.4)	2 (1.7)	-	
HOCMP	7 (4.9)	5 (4.2)	2 (8.3)	
Unknown	3 (2.1)	2 (1.7)	1 (4.2)	
**INTERMACS patient profile, n (%)**				0.129
INTERMACS 1	23 (16.2)	20 (17.0)	3 (12.5)	
INTERMACS 2	70 (49.3)	61 (51.7)	9 (37.5)	
INTERMACS 3	31 (21.8)	25 (21.2)	6 (25.0)	
INTERMACS 4	10 (7.0)	8 (6.8)	2 (8.3)	
INTERMACS 5–7	8 (5.6)	4 (3.4)	4 (16.7)	
**Number of inotropes, n (%)**				0.042
No	31 (21.8)	22 (18.6)	9 (37.5)	
Yes	111 (78.2)	96 (81.4)	15 (62.5)	
**Mechanical ventilation, n (%)**				0.425
No	103 (72.5)	84 (71.2)	19 (79.2)	
Yes	39 (27.5)	34 (28.8)	5 (20.8)	
**ECMO support, n (%)**	27 (19.0)	22 (18.6)	5 (20.8)	0.803

**Table 3:** Reasons for death. N: number; CAV: cerebrovascular accidents.

	Reason for Death	HVAD Group	HM3 Group
Death, n (%)		16 (14%)	3 (12%)
	CVA, n	4 (25%)	0
	Infection, n	4 (25%)	1 (33%)
	Multi-organ failure, n	6 (37%)	2 (67%)

## Data Availability

Original anonymized data can be supplied upon request.

## Abbreviations

CHD	Congenital heart disease
CMO	Cardiomyopathy
EACTS	European Association for Cardio-Thoracic Surgery
EUROMACS	European Registry for Patients with Mechanical Circulatory Support
ECMO	Extracorporeal membrane oxygenation
HM3	HeartMate 3
HVAD	HeartWare ventricular assist device
IC	Intracorporeal
IMACS	Interagency Registry for Mechanical Circulatory Support
LVAD	Left ventricular assist device
MCS	Mechanical circulatory support
RVAD	Right ventricular assist device
VAD	Ventricular assist device
